# Book Review

**Published:** 1912-12

**Authors:** 


**Children, their Care and Management.** By E. M. Brockbank, M.D. Vict., F.R.C.P. Pp. xii, 259. London: Henry Frowde, and Hodder & Stoughton. 1912. Price 3s. 6d. net.—This is one of the best books on the care and management of children we have ever read. It is packed full of valuable information and advice on every point the newly-qualified practitioner is expected to know. The language used is simple, and admits of no misunderstanding as to the author's meaning. Every page bears the impress of experience and common sense. We feel sure the book will prove of use to a large circle of readers.

